# Mapping of a responsible region for sex reversal upstream of *Sox9* by production of mice with serial deletion in a genomic locus

**DOI:** 10.1038/s41598-018-35746-0

**Published:** 2018-11-30

**Authors:** Yuya Ogawa, Miho Terao, Satoshi Hara, Moe Tamano, Haruka Okayasu, Tomoko Kato, Shuji Takada

**Affiliations:** 1 Department of Systems BioMedicine, National Research Institute for Child Health and Development, Tokyo, 157-8535 Japan; 2 Present Address: Tokyo Metropolitan Institute of Medical Science, Regenerative Medicine Project, Tokyo, 156-8506 Japan

## Abstract

*Sox9* plays critical roles in testis formation. By mapping four familial cases of disorders of sexual development, a 32.5 kb sequence located far upstream of *SOX9* was previously identified as being a commonly deleted region and named the XY sex reversal region (XYSR). To narrow down a responsible sequence in XYSR, we generated mutant mice with a series of deletions in XYSR by application of the CRISPR/Cas9 system, using a mixture of sgRNAs targeting several kilobase (kb) intervals in the region. When the whole XYSR corresponding sequence in mice was deleted in XY karyotype individuals, the mutation resulted in female offspring, suggesting that an expression mechanism of *SOX9*/*Sox9* through XYSR is conserved in human and mouse. Male-to-female sex reversal was found in mice with a 4.8 kb deletion. We identified a sequence conserved among humans, mice, and opossum, the deletion of which (783 bp) in mice resulted in male-to-female sex reversal. The sequence includes a recently reported critical gonad enhancer for *Sox9*. Although it cannot be concluded that the human sequence is responsible for XYSR, it is likely. This method is applicable for fine mapping of responsible sequences for disease-causing deletions especially with regard to rare diseases.

## Introduction

In most mammals, male sex is determined by the presence of the Y-linked sex-determining gene, *Sry* (sex-determining region Y)^1,2^. Transgenic mice containing *Sry* and XX karyotype are males^3^ and knockout of the *Sry* gene turns mice into females^4,5^. It has been assumed that the only function of SRY is up-regulation of *Sox9* (SRY-box 9), which encodes a transcription factor required for testis formation^6,7^. This occurs through binding of SRY to a specific *Sox9* enhancer, TESCO (testis-specific enhancer of *Sox9* core), located 11–13 kb upstream to *Sox9*^8^. However, TESCO is not the sole enhancer required for sex-determination, since TESCO deleted mice with an XY karyotype were male^9^.

Embryonic expression of *Sox9* is crucial for testis formation. In humans, haploinsufficiency of *SOX9* causes campomelic dysplasia (CD)^10,11^, which is characterized by congenital bowing and angulation of long bones with up to two-thirds of CD patients of 46,XY genotypic males presenting with a range of disorders of sexual development (DSD). In mice, testis differentiation is interrupted by homozygous deletion of *Sox9* in early XY gonads^12^, while mice with heterozygous deletion of *Sox9* in the whole body show histologically normal testes with skeletal malformations strongly resembling those in CD patients^13^. In addition, ectopic expression of *Sox9* in early female gonads results in XX sex reversal^7,14^.

It is likely that there are elements regulating *Sox9* expression in embryonic gonads other than TESCO, as studies have identified deletions located more than several hundred kilo base pairs (kb) upstream of the *SOX9* transcription start site in patients of 46,XY complete gonadal dysgenesis in the absence of CD^15,16^. In addition, chromosomal translocation breakpoints in CD cases with XY sex reversal are distributed from 50 kb to several 100 kb upstream of *SOX9*^17^. Such deleted sequences and breakpoints are located far upstream of the TESCO homologous sequence.

In 2015, Kim *et al*.^18^ identified a region that is deleted in four families with SRY-positive 46,XY DSD without skeletal phenotype approximately 600 kb upstream of *SOX9*. This region is 32.5 kb and designated as the XY sex reversal region (XYSR). The authors assumed that there could be one or more enhancers essential for activation of *SOX9* in the early male gonad^18^. As such, in order to identify an enhancer, it is necessary to narrow down the responsible sequence within XYSR.

With the rise of genome editing technology using RNA-guided clustered regularly interspaced short palindrome repeat-associated Cas9 nuclease (CRISPR/Cas9)^19,20^, production of mutant mice has become widely accessible. In the CRISPR/Cas9 system, a complex of single-guide RNAs (sgRNAs), which recognize a target sequence, and Cas9 induce a double strand break (DSB); subsequently the generated lesion is repaired by non-homologous end joining (NHEJ). NHEJ is an error-prone mechanism and insertions and deletions (indels) can be introduced. Although genome editing technology was originally used to introduce small indels into a genome, methods were recently developed to enable generation of various mutant mice. For instance, deletion can be induced by utilization of a pair of sgRNAs, targets of which are set at both ends of the desired deletion sequence, since the lesions at the edge of the sgRNA spanned sequence can be connected by NHEJ^21^.

In this study, we aimed to narrow down the responsible sequence with XYSR using mice as a model, by application of the CRISPR/Cas9 system.

## Results

### Estimation of mouse genomic sequence syntenic to human XYSR sequence

**Figure 1:**
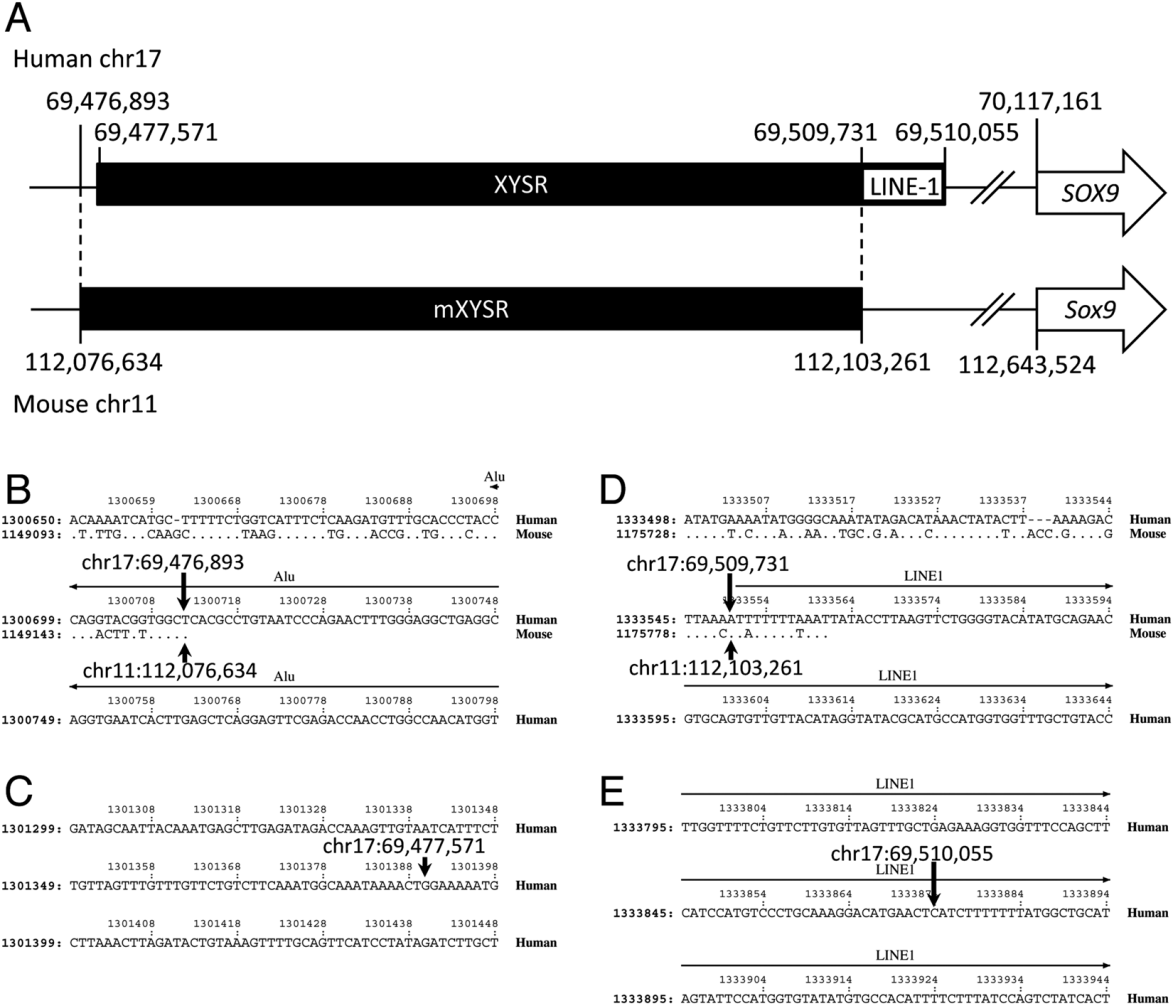
Identification of mXYSR. (**A**) Schematic representation of the XYSR/mXYSR region. Black bar, black box, white box and arrow indicate the genome, XYSR/mXYSR, LINE-1 element and *SOX9*/*Sox9*. Positions are shown with vertical bars and numbers. (**B**–**E**) Results of Pipmaker analysis. The centromeric termini of mXYSR (**B**) and XYSR (**C**) and the telomeric termini of mXYSR (**D**) and XYSR (**E**) are shown. Human and mouse sequences are shown at the top and bottom, respectively. Conserved nucleotides are indicated by dots on the mouse sequence. Hyphens indicate sequences that only exist in the human or mouse. In the non-conserved regions, only the human sequence is shown. Numbers at the top and left are nucleotide positions of query sequences used for the analysis. Horizontal arrows show the area of repetitive sequences. Arrows pointing up and down indicate the positions of edges of XYSR and mXYSR, respectively.

In humans, the XYSR sequence corresponds to chr17:69,477,571-69,510,055 (32,485 bp) of the draft human genome sequence^18^. Since human and mouse genomes contain the same order of genes, *KCNJ2*/*Kcnj2-SOX9*/*Sox9*-*SLC39A1*/*Slc39a1*, the sequence between *KCNJ2*/*Kcnj2* and *SLC39A1*/*Slc39a1* of human/mouse sequences were compared. Human and mouse sequences used were chr17:68,176,182- 70,642,085 and chr11:110,927,479-113,106,168, respectively. PipMaker analysis showed that the first 500 base pairs (bp) of XYSR has no homology to the mouse sequence. The closest sequence position hit to the mouse sequence of human chr17:69,477,571 was chr17: 69,476,893, which hit to mouse chr11:112,076,634 (Fig. 1). RepeatMasker analysis showed that the last 324 bp (chr17: 69,509,732-69,510,055) of XYSR is a LINE/L1 element and chr17:69,509,731 aligns to chr11:112,103,261 of the mouse genomic sequence. From this result, we predict the position of a possible XYSR region in mouse (mXYSR) is chr11:112,076,635-112,103,261 (26,627 bp) (Fig. 1).

### Series of mutant mice generation

**Figure 2:**
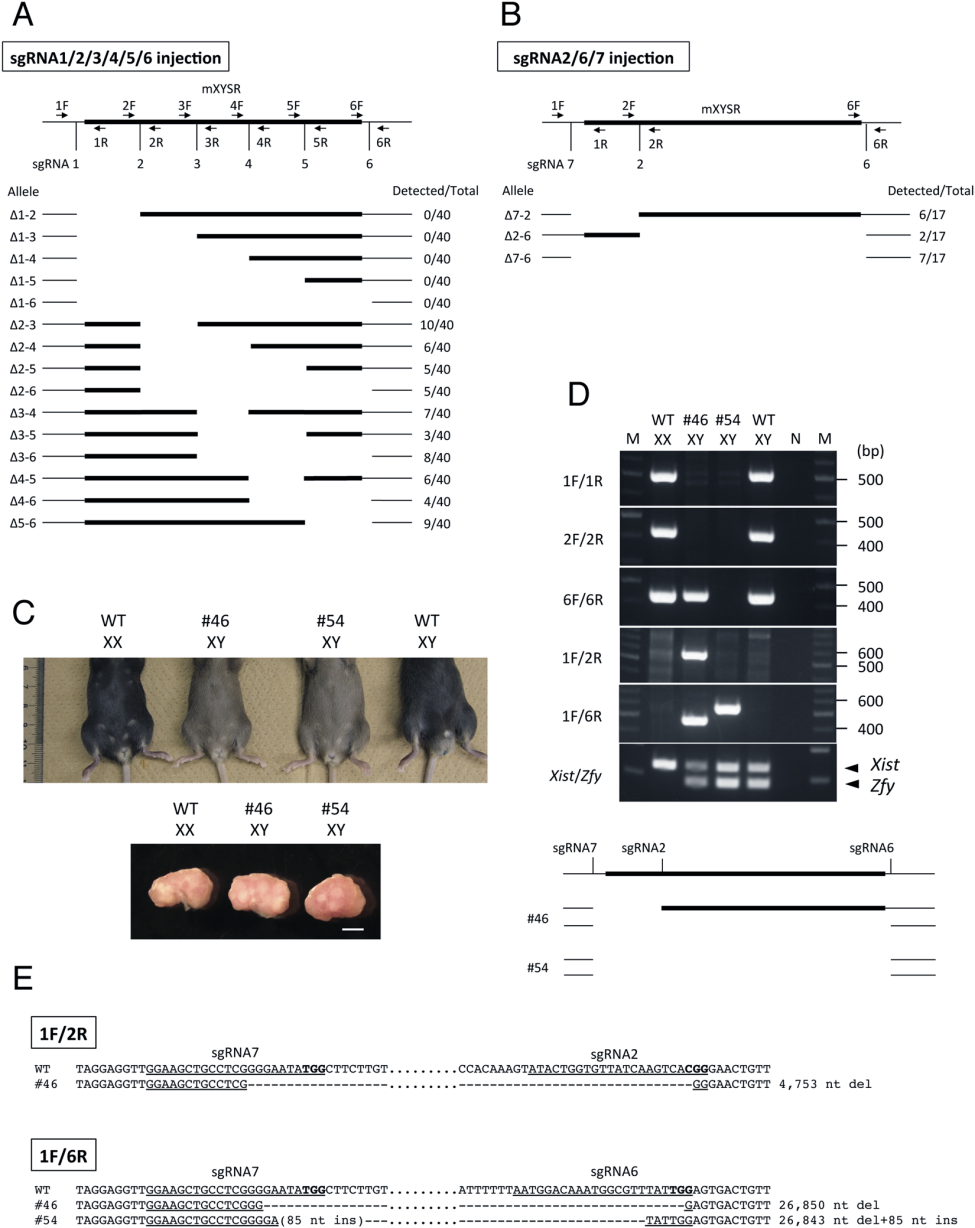
Generation of mutant mice using series deletion. (**A**,**B**) Schematic representation of series deletion made using sgRNA1-6 (**A**) sgRNA2/6/7 (**B**). Black bar and black box indicate genome and mXYSR, respectively. PCR primers and sgRNAs are demarcated with arrows and vertical bars. Allele names and numbers of mice are shown at left and right, respectively. WT: wild-type. (**C**) Phenotype of mutant mice. External and internal genitalia are shown at top and bottom, respectively. Genotype or ID of mice, and karyotype are shown at top of each photo. WT: wild type. Scale bar: 1 mm. (**D**) Electrophoresis of PCR genotyping (top) and schematic representation of deleted regions of mutant mice (bottom). Genotypes or IDs of mice, and karyotypes are shown at top. Primer sets are indicated to the left. Sizes of DNA markers are shown on the right. Positions of *Xist* and *Zfy* PCR products are indicated with arrowheads at right. Full agarose gel images of (**D**) are shown in Supplementary Fig. S1. M: DNA marker; N: negative control; WT: wild type. In the bottom figure, the black bar and box are similar to those described for (**A**). Positions of sgRNAs and mouse IDs are designated at the top and left, respectively. (**E**) Nucleotide sequences of deleted alleles. Sequences of PCR products amplified with primer pairs 1 F/2 R and 1 F/6 R are shown. The pairs of PCR primers used are shown with boxes. Mouse IDs and genotypes are shown on the left and right, respectively. Underlined are the positions of each target sequence of sgRNAs. A parenthesis shows unexpected nucleotide insertion. Bold letters indicate PAM sequence. Hyphens: deleted nucleotides; ins: insertion; del: deletion.

Production of mutant mice with deletion of whole or parts of the mXYSR region was carried out using the CRISPR/Cas9 system. To this end, we generated six sgRNAs set inside and outside the mXYSR region (Fig. 2A). The six sgRNAs were mixed together with Cas9 mRNA and injected into fertilized eggs. Forty F_0_ pups were obtained and genotype analysis of these pups showed that various alleles were generated between sgRNA2 and sgRNA6 and sgRNA1 did not work (Fig. 2A, Table S1). Estimated deletion sizes between sgRNAs are as follows; sgRNA2/sgRNA3: 5.5 kb; sgRNA2/sgRNA4: 12 kb; sgRNA2/sgRNA5: 18.2 kb; sgRNA2/sgRNA6: 22.1 kb; sgRNA3/sgRNA4: 6.5 kb;sgRNA3/sgRNA5: 12.7 kb; sgRNA3/sgRNA6: 16.6 kb; sgRNA4/sgRNA5: 6.2 kb; sgRNA4/sgRNA6: 10.1 kb; sgRNA5/sgRNA6: 4.0 kb. To obtain mutants with deletions of sgRNA1-sgRNA2 and sgRNA1-sgRNA6 (whole mXYSR) regions, we designed sgRNA7 and injected sgRNA2/sgRNA6/sgRNA7 with Cas9 mRNA into fertilized eggs (Fig. 2B). Seventeen F_0_ pups were obtained and 13 pups contained mutant alleles with sgRNA7-sgRNA2 (4.8 kb), sgRNA2-sgRNA6 (22.2 kb) or sgRNA7-sgRNA6 deletion (26.9 kb) (Fig. 2B, Table S2).

Next, we determined phenotypic and genotypic sexes in all obtained F_0_ pups injected with sgRNAs and Cas9. The result showed that sex assayed by external genitalia observation, and chromosome constitution, identified by PCR sexing, were identical in all pups injected with sgRNA1-sgRNA6 and Cas9. On the other hand, the same analysis in pups injected with sgRNA2/6/7 and Cas9 showed that sex indicated by external genitalia and chromosome constitution by PCR sexing were identical in most pups except 3 (#46, #48, and #54), which were phenotypically female with XY karyotype (Fig. 2C,D). The gonads of #46 and #54 were indistinguishable from wild type females and presented as ovaries (Fig. 2C). These male-to-female sex-reversed pups had two types of genotype, one had the sgRNA7-sgRNA6 deletion only (#54) and the other (#46 and #48) had sgRNA7-sgRNA2 and sgRNA7-sgRNA6 deletions (Fig. 2D,E). We concluded that mXYSR is required for male development where the XYSR and sgRNA7-sgRNA2 regions are responsible for sex reversal. The human sequence corresponding to mouse sgRNA7-sgRNA2 region (chr11:112,076,427-112,081,194) is chr17:69,476,660-69,482,891 (6,232 bp).

### Identification of the responsible region of mXYSR between sgRNA7-sgRNA2

**Figure 3:**
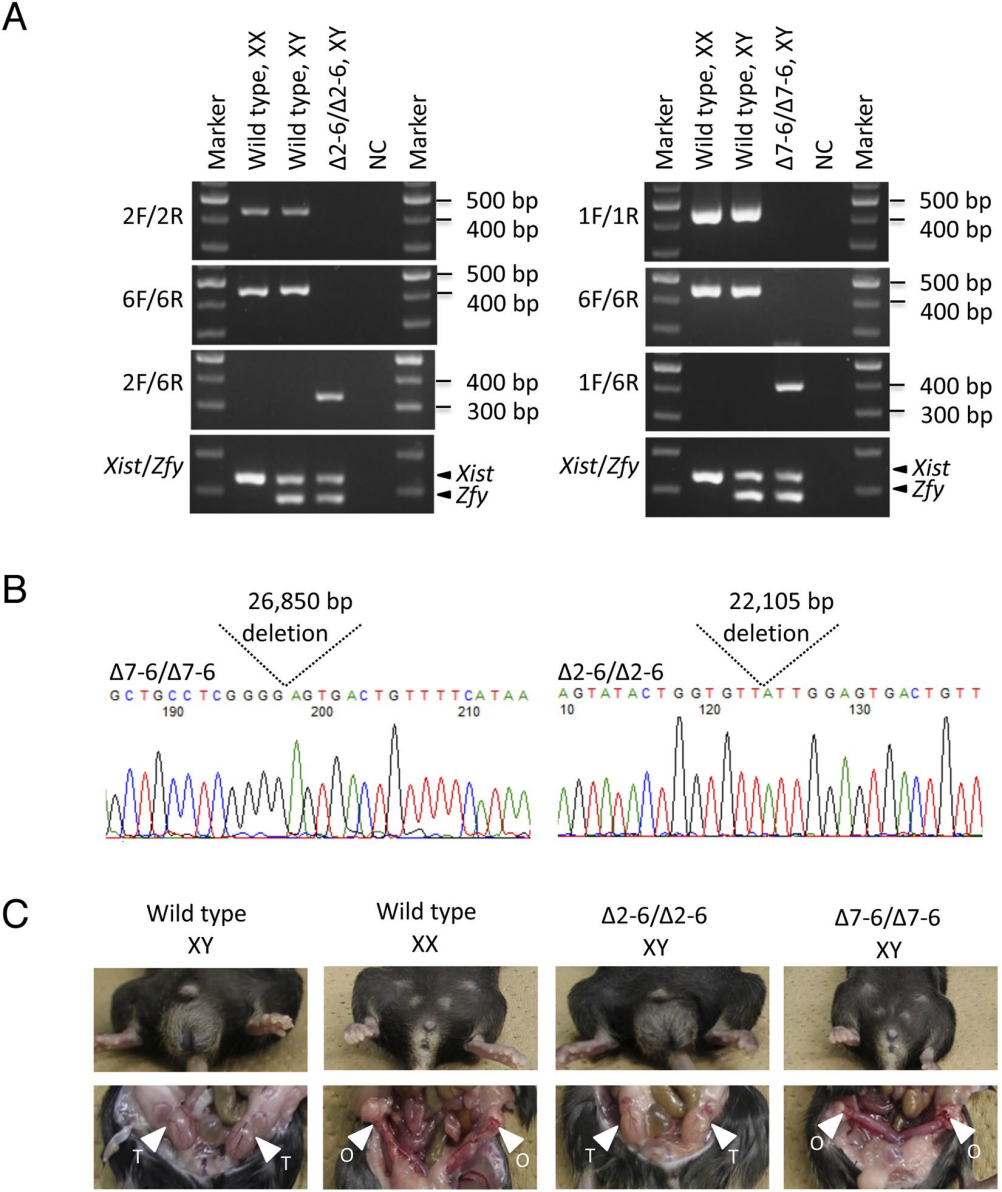
Phenotypic analysis of homozygous mutants. (**A**) Electrophoresis of PCR genotyping. Genotypes and karyotypes are shown at the top. Primer sets are indicated to the left. Positions of *Xist* and *Zfy* PCR products are indicated with arrowheads at the right. Sizes of DNA markers are shown on the right. NC: negative control. Full agarose gel images of left and right part of (**A**) are shown in Supplementary Figs S2 and S3, respectively. (**B**) Sequences of deleted alleles are shown with electropherograms. Genotypes are indicated at top left. Positions of deleted sequences are indicated with dotted lines. The deleted lengths are shown between dotted lines. (**C**) Phenotype of mutant mice. Genotypes and karyotypes are shown at top. Testis and ovary are indicated with arrowheads labeled T and O, respectively.

Next, we generated mutant mice with serial deletions by mating the F_1_ generation. Ten F_0_ mice injected with sgRNA1-6 were crossed with wild-type C57BL/6 and we obtained 80 F_1_ pups in total. Genotyping analysis showed that heterozygous F_1_ pups carrying serial deletions between sgRNA2 and sgRNA6 were successfully obtained, indicating that serial deletion alleles observed in F_0_ mice can be isolated in the F_1_ generation by back-crossing (Table S3). Similarly, two F_0_ mice injected with sgRNA2/6/7 were crossed with wild type and isolated F_1_ heterozygous mutants carrying the sgRNA7-6 deletion (26,850 bp chr11:112,076,444-112,103,293) (Table S3). F_1_ heterozygous males and females containing the same mutation were mated to generate F_2_ homozygous mutants (Fig. 3B). As expected, XY individuals of homozygous mutants with the sgRNA7-sgRNA6 deletion showed female type external and internal genitalia (Fig. 3C). XY individuals, homozygous mutants of sgRNA2-sgRNA6 (22,105 bp deletion, chr11:112,081,183-112,103,287), had male type external and internal genitalia, suggesting that the sgRNA2-sgRNA6 region is not critical for testis development.

### Identification of evolutionary conserved sequences in XYSR

**Figure 4:**
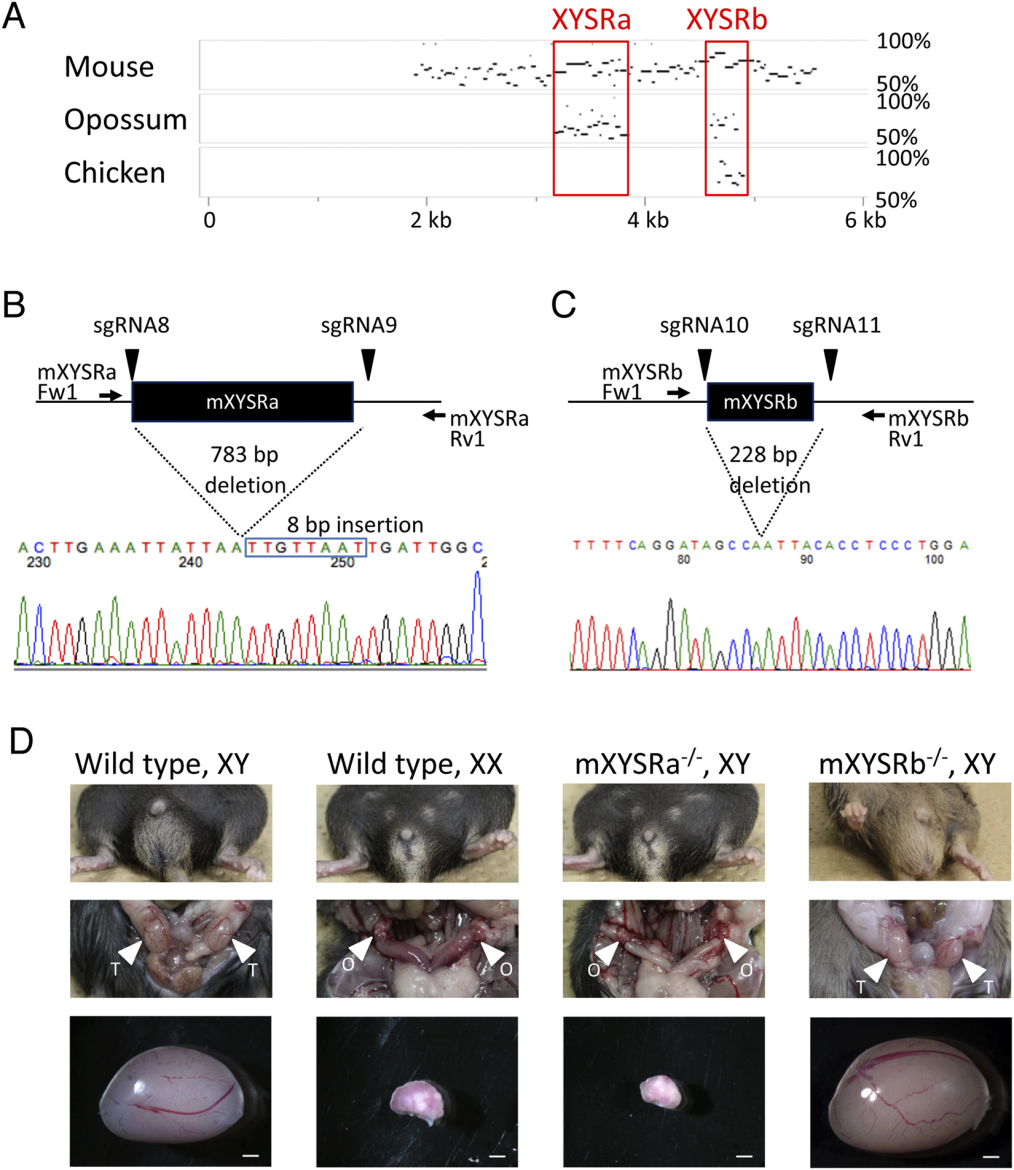
Identification of mXYSRa as the responsible sequence of mXYSR. (**A**) A result of MultiPipMaker analysis. Mouse, opossum, and chicken sequences homology at 50% or more to human XYSR (shown horizontally) are plotted by MultiPipMaker. Positions in XYSR are shown at the bottom. Species and homology levels are indicated at the left and right, respectively. (**B**,**C**) Results of genome editing of the mXYSRa and mXYSRb. Positions of sgRNAs and PCR primers are shown with arrowheads and arrows, respectively, on genome sequences (black lines). Black boxes indicated mXYSRa/mXYSRb. Sequences of deleted alleles are shown with electropherograms at the bottom. Positions of deleted sequences are indicated with dotted lines. The deleted lengths are shown between dotted lines. The inserted sequence is indicated with a blue box. Electropherogram of mXYSRa is shown after the original image was reverse complemented using FinchTV software. (**D**) Phenotypes of mXYSRa/mXYSRb homozygous deleted mice. Extra genitalia are shown with genotype. Testis and ovary are indicated with arrowheads labeled T and O, respectively. Scale bar: 1 mm.

To further narrow down the responsible sequence for XYSR, we searched for evolutionally conserved sequence(s) in human chr17:69,476,660-69,482,891, as important regulatory sequences tend to be conserved among species. For this purpose, draft genomic sequences between *Kcnj2* and *Slc39a11* of mouse, opossum, and chicken were collected (where successive undetermined sequences (N)_n_ in both chicken and opossum were substituted with N and the opossum sequence was reverse complemented in order to align the orientation) and analyzed by MultiPipMaker using the repeat masked human sequence as query. The mouse, opossum, and chicken sequences used were chr11: 110,927,479-113,106,168, chr2:218,051,485-222,048,290, and chr18:8,211,892-8,929,309, respectively. As shown in Fig. 4A, two evolutionarily conserved sequences were identified, one conserved in human, mouse, and opossum, and the other in human, mouse, opossum, and chicken. We label the human/mouse former sequence XYSRa/mXYSRa and latter XYSRb/mXYSRb, respectively. Positions in mouse and human are as follows: XYSRa: chr17:69,480,702-69,481,532; mXYSRa: chr11:112,078,498-112,079,208; XYSRb: chr17:69,482,233-69,482,421; and mXYSRb: chr11:112,080,455-112,080,649.

Next, we generated mXYSRa and mXYSRb deletion mice using sgRNA8/sgRNA9 and sgRNA10/sgRNA11, respectively, using CRISPR/Cas9. Both sgRNA8 and sgRNA9 are targeted outside mXYSRa and sgRNA10/sgRNA11 of mXYSRb (Fig. 4B,C). PCR genotyping analysis showed that alleles with a 783 bp deletion (chr11:112,078,499-112,079,281) including mXYSRa and an insertion of 8 bp of an unidentified sequence (Fig. 4B), and with a 228 bp deletion (chr11:112,080,452-112,080,679) including mXYSRb were identified (Fig. 4C). Homozygous mutants of mXYSRb with XY karyotypes had male external and internal genitalia, whereas mXYSRa with XY karyotypes female, indicating mXYSRa is the responsive sequence of mXYSR (Fig. 4D).

## Discussion

We produced a series of mice with serially deleted alleles from the sgRNA2 to the sgRNA6 region by single microinjection of mixed sgRNAs and Cas9 into fertilized eggs. This methodology will be useful for fine mapping of a disease-causing genomic sequences especially in the case of rare diseases. It is also applicable for promoter analysis. To identify critical sequences in promoters, usually a series of plasmids containing various lengths of promoter fragments with a reporter is transfected in to cultured cells and the reporter activity assayed. This is an easy, quick, and cost effective method; however, the results obtained may not always reflect the *in vivo* situation where there may not be a suitable culture condition, or a gene of interest is regulated by a cis-sequence element located at a distance requiring the entire genome to test. In such cases, production of a series of deletions in a genome using our method can present an alternative.

In the present study, we showed that deletion of the mouse sequence corresponding to XYSR caused male-to-female sex reversal, as observed in humans, suggesting that there is a common mechanism mediated by *Sox9* expression through the XYSR region; however, there is a difference between humans and mice, i.e., sex reversal was observed in both heterozygous and homozygous humans and mice, respectively. This difference can be explained by the required amount of *SOX9* for inducing testis from undifferentiated gonads being controlled by two chromosomes in humans and one chromosome in mice since testes are formed in the heterozygous *Sox9* knockout mouse and ovaries can be formed in heterozygous *SOX9* mutation CD patients.

We narrowed down the sequence responsible for XYSR in mice to 4.8 kb (sgRNA7-sgRNA2) by producing series of deletion alleles. It corresponds to approximately 6.2 kb in humans at the centromeric side of XYSR. At the moment, we cannot prove that the human 6.2 kb sequence is responsible for XYSR until XYSR is further narrowed down by mapping of an XY patient with disorder of sex development.

It is possible that there are one or more enhancers in the sgRNA7-sgRNA2 sequence for early male gonadal expression of *Sox9*. Homozygous XY mutant mice with sgRNA7-sgRNA2 deletions had female external genitalia, as with *Sry* KO mouse^4,5^, implying that the putative enhancer begins to function at early stages of testis formation. It may be also possible that there is a weak enhancer in the sgRNA2-sgRNA6 region; however, such an enhancer cannot be identified by production of a series of mutant mice as performed in the present study.

In this study, after identification of sgRNA7-sgRNA2 as a responsible sequence of mXYSR, we tried to further narrow it down and finally identified mXYSRa. To do this, we utilized evolutionary conservation-based screening instead of making mutant mice with a series of deletions in mXYSRa, in the same way as for the identification of mXYSRa. The reason why we did not adopt the second round of generation of mutants with a series of deletions in mXYSRa using a mixture of sgRNAs is that several kb of the target region would be too small; we empirically anticipated that most mice generated in this way would have whole mXYSRa deletion. We believe evolutionary conservation-based screening was a good choice for rapid identification of the responsible sequence since there are only two sequences, mXYSRa and mXYSRb, that have similarities among human, mouse and, opossum genomes. mXYSRa is conserved across the mammals tested but not in chicken, implying that mXYSRa functions through a mammalian-specific mechanism. Further refinement of the responsive sequence at a single nucleotide level may help to clarify the mechanisms in place.

Very recently, Gonen *et al*.^22^. reported Enh13 as a critical enhancer for *Sox9* expression in mouse embryonic gonads. They identified Enh13 based on open chromatin structure, and its deletion in mice resulted in male-to-female sex reversal. Since the entire sequence of Enh13 (557 bp) is in mXYSRa (711 bp), the phenotypes of deletion mice of Enh13 and mXYSRa are the same, suggesting that our strategy for identification of responsible sequences from large genomic regions is useful.

## Materials and Methods

### Bioinformatics

Draft genome sequences were obtained from the University of California, Santa Cruz (UCSC) genome browser (http://genome.ucsc.edu/). The genome assemblies used were February 2009 (GRCh37/hg19), July 2007 (NCBI37/mm9), October 2006 (Broad/monDom5), and 2006 (WUGSC 2.1/galGal3) for human, mouse, opossum, and chicken, respectively. Repeat sequences in the genomic sequence were identified using RepeatMasker (http://www.repeatmasker.org/cgi-bin/WEBRepeatMasker). Sequence homology search and alignment were performed using PipMaker and MultiPipMaker (http://pipmaker.bx.psu.edu/pipmaker/)^23^.

### Plasmids

Human codon optimized Cas9 (hCas9) and gRNA Cloning Vector were gifts from George Church (Addgene plasmid # 41815 and # 41824, respectively)^20^. The sgRNA-cloning vector was used, with some modifications as previously described^24^.

### Construction of sgRNA vectors

sgRNA sequences were cloned by inverse PCR using primers for sgRNA cloning (Table 1) and a modified sgRNA cloning vector as a template. The primers used were sgRNA1 Fw/sgRNA1 Rv, sgRNA2 Fw/sgRNA2 Rv, sgRNA3 Fw/sgRNA3 Rv, sgRNA4 Fw/sgRNA4 Rv, sgRNA5 Fw/sgRNA5 Rv, sgRNA6 Fw/sgRNA6 Rv, sgRNA7 Fw/sgRNA7 Rv, sgRNA8 Fw/sgRNA8 Rv, sgRNA9 Fw/sgRNA9 Rv, sgRNA10 Fw/sgRNA10 Rv, sgRNA11 Fw/sgRNA11 Rv for amplification of sgRNA1, sgRNA2, sgRNA3, sgRNA4, sgRNA5, sgRNA6, sgRNA7, sgRNA8, sgRNA9, sgRNA10 and sgRNA11, respectively. PCR products were digested with *Dpn*I (New England Biolabs Inc., Beverly, MA, USA) and transformed into DH5α cells, as previously described^24,25^. The sgRNAs sequences were verified by sequencing analysis.

**Table 1:** Primers used in this study.

Primer name	Sequence (5′ to 3′)
sgRNA1 Fw	AAGACAGTACTGACTACATgttttagagctagaaatagcaag
sgRNA1 Rv	aacATGTAGTCAGTACTGTCTTcggtgtttcgtcctttccac
T7sgRNA1	ttaatacgactcactataggAAGACAGTACTGACTACAT
sgRNA2 Fw	TACTGGTGTTATCAAGTCAgttttagagctagaaatagcaag
sgRNA2 Rv	aacTGACTTGATAACACCAGTAcggtgtttcgtcctttccac
T7sgRNA2	ttaatacgactcactataggTACTGGTGTTATCAAGTCA
sgRNA3 Fw	CCTCTCGGCACTACTTTAGgttttagagctagaaatagcaag
sgRNA3 Rv	aacCTAAAGTAGTGCCGAGAGGcggtgtttcgtcctttccac
T7sgRNA3	ttaatacgactcactataggCCTCTCGGCACTACTTTAG
sgRNA4 Fw	ATGTCTGTTGTATGACCAAgttttagagctagaaatagcaag
sgRNA4 Rv	aacTTGGTCATACAACAGACATcggtgtttcgtcctttccac
T7sgRNA	ttaatacgactcactataggATGTCTGTTGTATGACCAA
sgRNA5 Fw	TCACTTGATAATAGCATGAgttttagagctagaaatagcaag
sgRNA5 Rv	aacTCATGCTATTATCAAGTGAcggtgtttcgtcctttccac
T7sgRNA5	ttaatacgactcactataggTCACTTGATAATAGCATGA
sgRNA6 Fw	ATGGACAAATGGCGTTTATgttttagagctagaaatagcaag
sgRNA6 Rv	aacATAAACGCCATTTGTCCATcggtgtttcgtcctttccac
T7sgRNA6	ttaatacgactcactataggATGGACAAATGGCGTTTAT
sgRNA7 Fw	GAAGCTGCCTCGGGGAATAgttttagagctagaaatagcaag
sgRNA7 Rv	aacTATTCCCCGAGGCAGCTTCcggtgtttcgtcctttccac
T7sgRNA7	ttaatacgactcactataggGAAGCTGCCTCGGGGAATA
sgRNA8 Fw	AGACTTGAAATTATTAAGAgttttagagctagaaatagcaag
sgRNA8 Rv	aacTCTTAATAATTTCAAGTCTcggtgtttcgtcctttccac
T7sgRNA8	ttaatacgactcactataggAGACTTGAAATTATTAAGA
sgRNA9 Fw	ATAACAAACATTTACTGATgttttagagctagaaatagcaag
sgRNA9 Rv	aacATCAGTAAATGTTTGTTATcggtgtttcgtcctttccac
T7sgRNA9	ttaatacgactcactataggATAACAAACATTTACTGAT
sgRNA10 Fw	ACATTTTCAGGATAGCCATgttttagagctagaaatagcaag
sgRNA10 Rv	aacATGGCTATCCTGAAAATGTcggtgtttcgtcctttccac
T7sgRNA10	ttaatacgactcactataggACATTTTCAGGATAGCCAT
sgRNA11 Fw	GGTGTAATTGGCACCTTAGgttttagagctagaaatagcaag
sgRNA11 Rv	aacCTAAGGTGCCAATTACACCcggtgtttcgtcctttccac
T7sgRNA11	ttaatacgactcactataggGGTGTAATTGGCACCTTAG
sgRNA Rv	AAAAGCACCGACTCGGTGCC
mXYSR 1 F	CCCATGTACAGTTCACGCTTC
mXYSR 1 R	CAGCCCCATAATAAGCAAGG
mXYSR 2 F	TTCCCAGAGAAGGTGACTGAA
mXYSR 2 R	CAGTTGAGGAAGGCAACACA
mXYSR 3 F	TGATGGAATGCAATGGAAAA
mXYSR 3 R	ATGGCAGCACTTCAGGACTT
mXYSR 4 F	CCAGAACATATCCATCCATGC
mXYSR 4 R	TTCCAAGCCCATTGAGTTTC
mXYSR 5 F	CGGATACTGCTACCCCATTC
mXYSR 5 R	TGGGTCAGGTGTACCTCCTC
mXYSR 6 F	CCTTTGCTACCCAAACCTCA
mXYSR 6 R	TTTGTGCGCAGACTATCAGG
mXYSRa Fw1	AATCACAAAAGGCACTGAGG
mXYSRa Rv1	ATTCAATCAACAGCTATACG
mXYSRb Fw1	AGCCTAGAACTTTCAGTGGG
mXYSRb Rv1	CGATTGGTGAATCCTGACTC
T7Cas9 Fw	TAATACGACTCACTATAGGGAGAATGGACAAGAAGTACTCCATTGG
Cas9 Rv	TCACACCTTCCTCTTCTTC

### RNA synthesis by *in vitro* transcription reaction

T7 RNA promoter sequence was added by PCR amplification of the sgRNAs using primers pair of T7sgRNA1 and sgRNA Rv for sgRNA1, T7sgRNA2 and sgRNA Rv for sgRNA2, T7sgRNA3 and sgRNA Rv for sgRNA3, T7sgRNA4 and sgRNA Rv for sgRNA4, T7sgRNA5 and sgRNA Rv for sgRNA5, T7sgRNA6 and sgRNA Rv for sgRNA6, T7sgRNA7 and gRNA Rv for sgRNA7, T7sgRNA8 and sgRNA Rv for sgRNA8, T7sgRNA9 and sgRNA Rv for sgRNA9, T7sgRNA10 and sgRNA Rv for sgRNA10, T7sgRNA11 and sgRNA Rv for sgRNA11 (Table 1). To add the T7 RNA promoter sequence to the hCas9 sequence, the primers T7 Cas9 Fw and Cas9 Rv (Table 1) were used for amplification of the hCas9 expression plasmid. PCR products were purified using a PCR purification kit (QIAGEN GmbH, Hilden, Germany) and quantified using a NanoDrop ND-1000 Spectrophotometer (Thermo Fisher Scientific, Waltham, MA USA). *In vitro* transcription was performed using mMESSAGE/mMACHINE T7 Kit (Ambion, Austin, TX, USA), followed by purification using MEGAclear RNA Purification Kit (Ambion) according to the manufacturer’s instructions.

### Microinjection

Superovulation was induced in F_1_ female C57BL/6 × DBA2 (B6D2F1) cross mice by intraperitoneal administration of 5 IU of pregnant mare’s serum gonadotropin and human chorionic gonadotropin according to standard procedures. Fertilized eggs at the pronucleus stage were collected in M2 medium from superovulated B6D2F1 females crossed with B6D2F1 males. RNA concentrations used for microinjection were 1 µg/µl in total (142.8 ng/µl each) for injection of sgRNA1/2/3/4/5/6 and Cas9, 1 µg/µl in total (250 ng/µl each) for injection of sgRNA2/6/7 and Cas9, 1 µg/µl in total (333 ng/µl each) for injection of sgRNA8 /9 and Cas9, and 1 µg/µl in total (333 ng/µl each) for injection of sgRNA10/11 and Cas9. The RNA mix was microinjected into the cytoplasm of zygotes using a FemtoJet microinjector (Eppendorf, Westbury, NY, USA). The microinjected embryos were incubated in KSOM medium at 37 °C for one day. The embryos were allowed to develop to the two-cell stage and were transferred into pseudopregnant ICR female mice. All mice were purchased from a local vender (the Sankyo Labo Service Corporation, Tokyo, Japan). Animal protocols were approved by the Animal Care and Use Committee at the National Research Institute for Child Health and Development. All experiments were conducted in accordance with approved animal protocols.

### Genotyping

Genomic DNA was extracted from the tail or fingertips of newborn pups. The genomic regions around the sgRNAs were amplified by PCR using BIOLINE Taq (BioLine, London, UK), GoTaq Green Mix (Promega Corp., Madison, WI, USA) or KOD FX Neo (Toyobo Co., Ltd, Osaka, Japan) using sets of 1 F/1 R, 2 F/2 R, and 1 F/2 R for ∆1-2 and ∆7-2; 1 F/1 R, 3 F/3 R, and 1 F/3 R for ∆1-3; 1 F/1 R, 4 F/4 R, and 1 F/4 R for ∆1-4; 1 F/1 R, 5 F/5 R, and 1 F/5 R for ∆1-5; 1 F/1 R, 6 F/6 R, and 1 F/6 R for ∆1-6; 2 F/2 R, 3 F/3 R, and 2 F/3 R for ∆2-3; 2 F/2 R, 4 F/4 R, and 2 F/4 R for ∆2-4; 2 F/2 R, 5 F/5 R, and 2 F/5 R for ∆2-5; 2 F/2 R, 6 F/6 R, 2 F/6 R for ∆2-6; 3 F/3 R, 4 F/4 R, and 3 F/4 R for ∆3-4; 3 F/3 R, 5 F/5 R, and 3 F/5 R for ∆3-5; 3 F/3 R, 6 F/6 R, and 3 F/6 R for ∆3-6; 4 F/4 R, 5 F/5 R, and 4 F/5 R for ∆4-5; 4 F/4 R, 6 F/6 R, and 4 F/6 R for ∆4-6; 5 F/5 R, 6 F/6 R, and 5 F/6 R for ∆5-6; mXYSRa Fw1/mXYSRa Rv1 for mXYSRa; mXYSRb Fw1/mXYSRb Rv1 for mXYSRb (Table 1). The PCR products were subject to direct sequencing analysis after ExoSAP-IT (Affymetrix Inc., Santa Clara, CA, USA) treatment. The genotypes were determined from peaks of electropherograms. In cases where overlapping peaks or no-overlapping peaks with insertion and or deletion when compared with wild type sequence were observed around the sgRNA target sequences, the genotype was considered as mutant. When the wild type sequence without any insertion or deletion was observed, the genotype was considered as wild type. The genomic regions spanning inter-sgRNAs sequences were amplified by PCR using BIOLINE Taq (BioLine, London), GoTaq Green Mix (Promega,) or KOD FX Neo (Toyobo) using primer sets described in Fig. 1. The PCR products were separated by 2.5% agarose gel electrophoresis. If a band was observed, it was considered to be a deleted allele. For determination of the deleted sequences by nucleotide level, the PCR products were cloned into a pGEM-T Easy Vector (Promega Corp.) and subject to sequencing analysis.

Genotypes and deleted sequences were identified by agarose gel electrophoresis or direct sequence of the PCR product.

PCR sexing was performed using primer mix for Y-linked *Zfy* and X-linked *Xist*^26^ and BIOLINE Taq or GeneAmp Fast PCR Master Mix (2×) (Thermo Fisher Scientific).

### Mutant phenotype analysis

F_1_ generation was produced by mating F_0_ with wild type C57BL/6. Mutants were maintained by mating with wild type C57BL/6. To generate homozygous mutants, heterozygous mutants of the same generation were crossed. Phenotypes of F_0_ (series of mutant mice generation), F_2_ (sgRNA7-6 deletion and sgRNA2-6 deletion), N_3_F_2_ (mXYSRa deletion) and F_3_ mutant mice (mXYSRb deletion) were analyzed at 4–10 weeks. As a parameter of sexual differentiation, shape of external genitalia and existence of nipples were examined.

## Electronic supplementary material

Supplementary figures

Supplementary tables
