# Supplementary material for: Mapping of a responsible region for sex reversal upstream of Sox9 by production of mice with serial deletion in a genomic locus
Source: Sci Rep. 2018 Nov 30;8:17514. doi: 10.1038/s41598-018-35746-0 (PMC6269501; doi:10.1038/s41598-018-35746-0)
Supplement: Supplementary file 1 — Supplementary figures [file 41598_2018_35746_MOESM1_ESM.pdf]

Supplementary figures

Mapping of a responsible region for sex reversal upstream of *Sox9* by production of mice with serial deletion in a genomic locus

Yuya Ogawa[#, 1], Miho Terao[#, 1], Satoshi Hara[#, 1], Moe Tamano[1], Haruka Okayasu[1], Tomoko Kato[§,1] and Shuji Takada[*, 1]

[1] Department of Systems BioMedicine, National Research Institute for Child Health and Development, Tokyo 157-8535, Japan

[#]These authors contributed equally to this work.

[§]Present address: Tokyo Metropolitan Institute of Medical Science, Regenerative Medicine Project, Tokyo 156-8506, Japan

*Corresponding author: Shuji Takada (takada-s@ncchd.go.jp, 2-10-1 Okura, Setagaya, Tokyo 157-8535, Japan, Tel & Fax: +81-3-3417-2498).

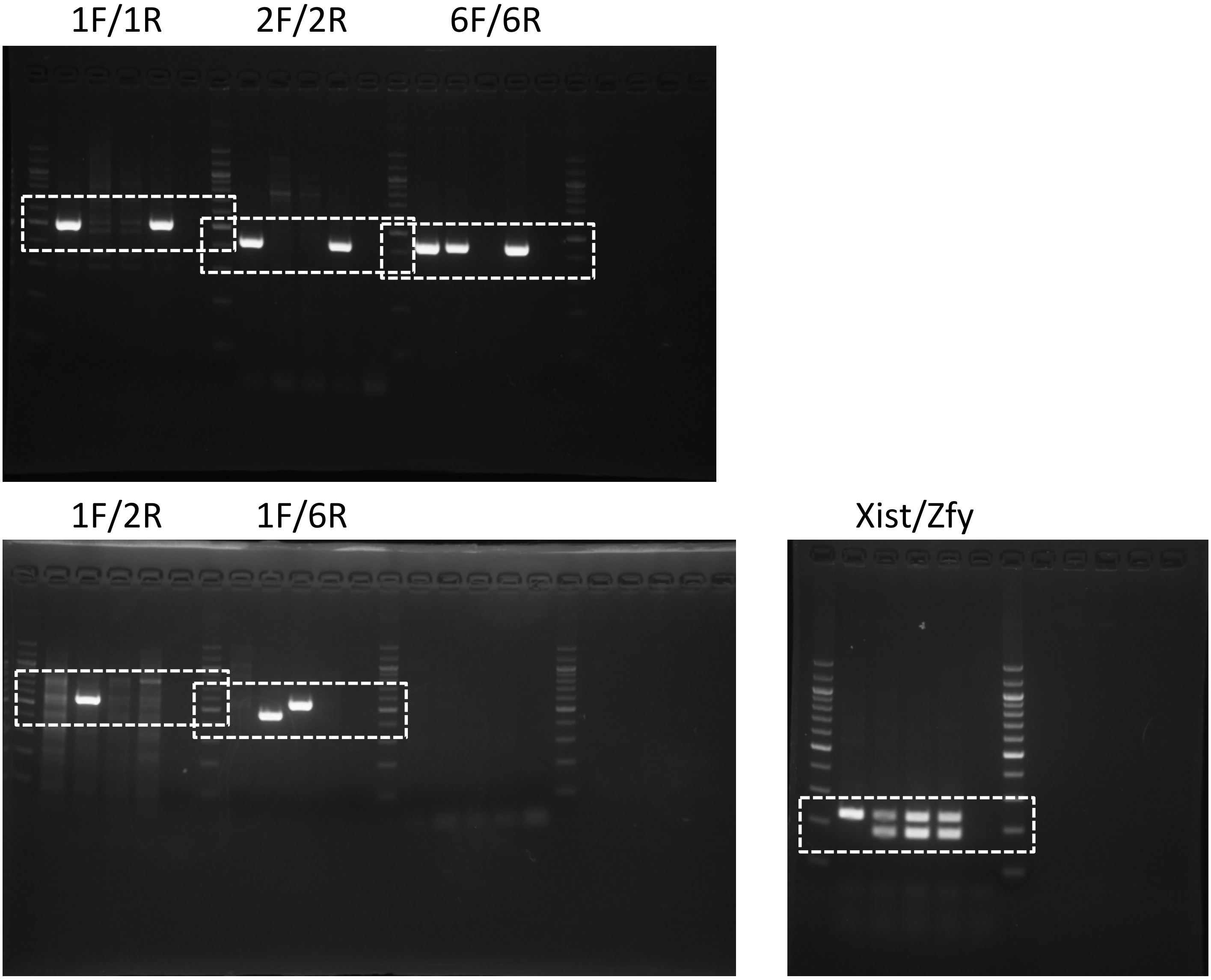

Supplementary Figure S1. Full agarose gel images of Figure 2D. Boxes with white dashed line indicate the cropped parts in the Figure 2D. Primers used are indicated at top of the images.

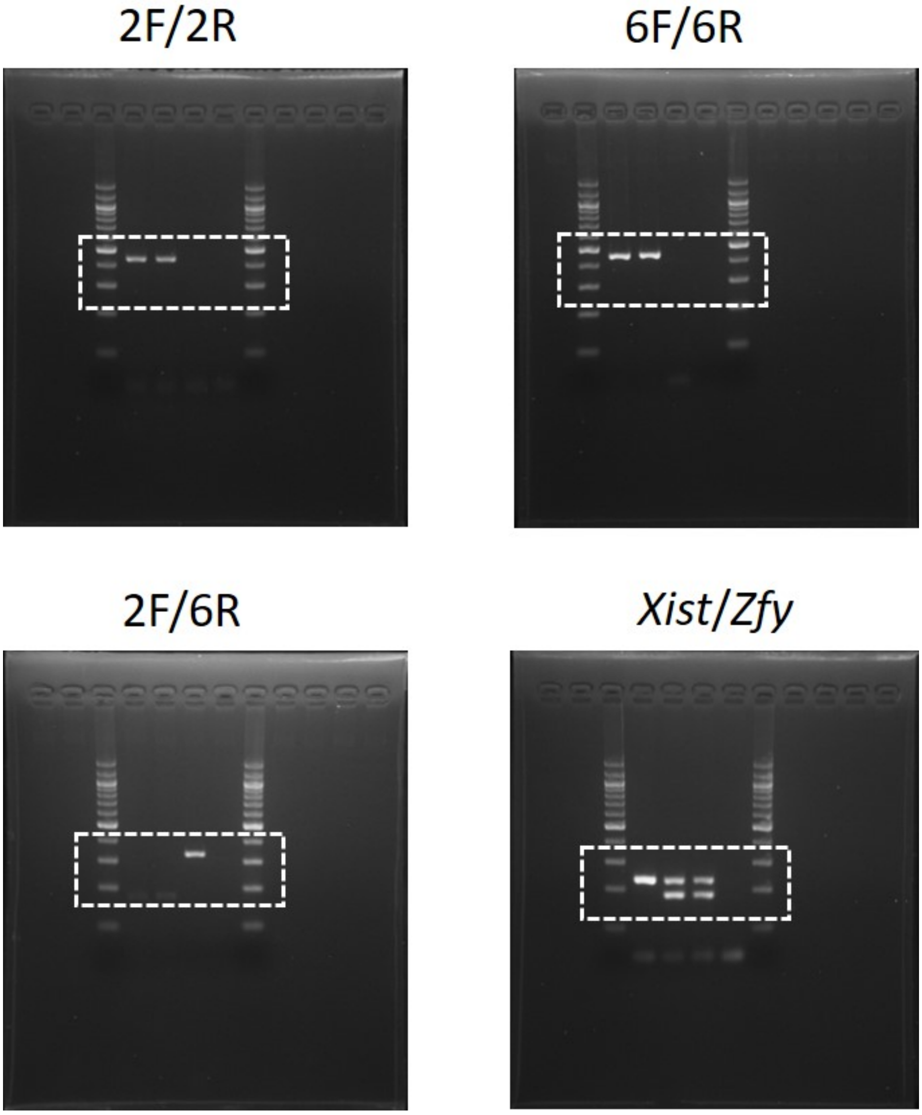

Supplementary Figure S2. Full agarose gel images of the left part of Figure 3A . Boxes with white dashed line indicate the cropped parts in the Figure 3A. Primers used are indicated at top of the images.

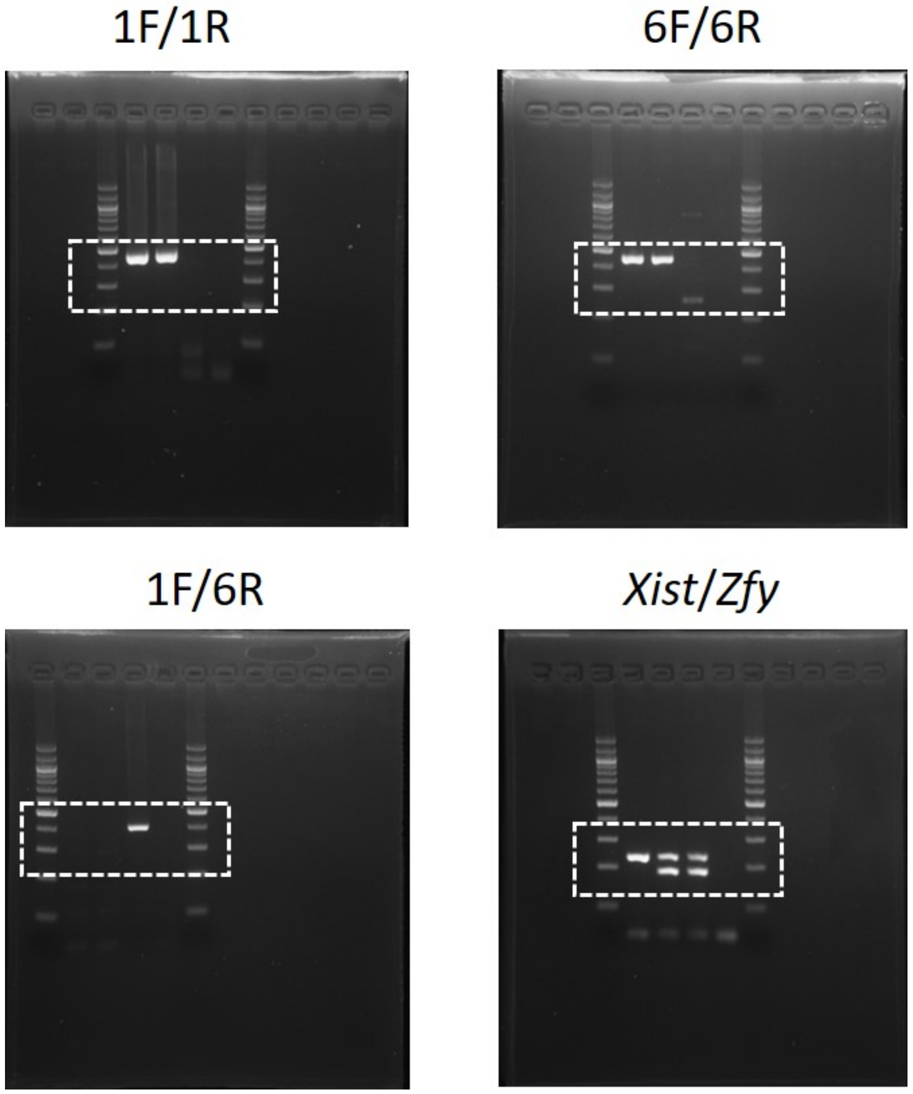

Supplementary Figure S3. Full agarose gel images of the right part of Figure 3A . Boxes with white dashed line indicate the cropped parts in the Figure 3A. Primers used are indicated at top of the images.
