# Supplementary material for: Mapping of a responsible region for sex reversal upstream of Sox9 by production of mice with serial deletion in a genomic locus
Source: Sci Rep. 2018 Nov 30;8:17514. doi: 10.1038/s41598-018-35746-0 (PMC6269501; doi:10.1038/s41598-018-35746-0)
Supplement: Supplementary file 2 — Supplementary tables [file 41598_2018_35746_MOESM2_ESM.pdf]

Supplementary tables

Mapping of a responsible region for sex reversal upstream of *Sox9* by production of mice with serial deletion in a genomic locus

Yuya Ogawa[#, 1], Miho Terao[#, 1], Satoshi Hara[#, 1], Moe Tamano[1], Haruka Okayasu[1], Tomoko Kato[§,1] and Shuji Takada[*, 1]

[1] Department of Systems BioMedicine, National Research Institute for Child Health and Development, Tokyo 157-8535, Japan

[#]These authors contributed equally to this work.

[§]Present address: Tokyo Metropolitan Institute of Medical Science, Regenerative Medicine Project, Tokyo 156-8506, Japan

*Corresponding author: Shuji Takada (takada-s@ncchd.go.jp, 2-10-1 Okura, Setagaya, Tokyo 157-8535, Japan, Tel & Fax: +81-3-3417-2498).

Table S1. Genotypes of F_0 mice obtained by injection of sgRNA1/2/3/4/5/6.

Mouse ID	Karyotype	Phenotypic sex	Indel						Deletion														
			F1/R1	F2/R2	F3/R3	F4/R4	F5/R5	F6/R6	F1/R2 (Δ1-2)	F1/R3 (Δ1-3)	F1/R4 (Δ1-4)	F1/R5 (Δ1-5)	F1/R6 (Δ1-6)	F2/R3 (Δ2-3)	F2/R4 (Δ2-4)	F2/R5 (Δ2-5)	F2/R6 (Δ2-6)	F3/R4 (Δ3-4)	F3/R5 (Δ3-5)	F3/R6 (Δ3-6)	F4/R5 (Δ4-5)	F4/R6 (Δ4-6)	F5/R6 (Δ5-6)
#1	XX	Female	WT	indel	ND	ND	ND	indel						○						○		○	
#2	XX	Female	WT	indel	indel	indel	WT	WT										○					○
#3	XY	Male	WT	indel	ND	ND	ND	ND															
#4	XY	Male	WT	indel	ND	ND	ND	WT															
#5	XX	Female	WT	ND	indel	indel	indel	WT															
#6	XY	Male	WT	ND	ND	ND	ND	ND						○								○	
#7	XY	Male	WT	indel	ND	ND	ND	ND						○			○						○
#8	XX	Female	WT	ND	ND	ND	ND	WT						○	○						○		
#9	XY	Male	WT	ND	ND	indel	indel	ND												○			○
#10	XY	Male	WT	indel	indel	indel	WT	WT						○					○	○			○
#11	XX	Female	WT	indel	indel	indel	indel	ND												○			
#12	XY	Male	WT	ND	indel	ND	WT	WT															
#13	XY	Male	WT	indel	indel	indel	WT	ND							○					○			
#14*	XX	Female	ND	ND	ND	ND	ND	ND															
#15	XY	Male	WT	indel	ND	indel	WT	WT													○		
#16	XY	Male	WT	ND	ND	ND	ND	ND							○		○						
#17	XX	Female	WT	indel	indel	ND	WT	ND											○				
#18	XY	Male	WT	indel	ND	ND	WT	ND															
#19	XX	Female	WT	ND	ND	ND	ND	ND									○						
#20	XX	Female	WT	ND	ND	ND	ND	ND									○						
#21	XY	Male	WT	ND	ND	ND	ND	WT								○	○						
#22	XX	Female	WT	indel	indel	ND	indel	indel										○					
#23	XY	Male	WT	indel	ND	ND	indel	indel							○	○		○	○				○
#24	XX	Female	WT	indel	indel	indel	indel	WT						○									
#25	XX	Female	WT	indel	ND	ND	ND	ND														○	
#26	XY	Male	WT	ND	ND	ND	ND	WT								○							○
#27	XX	Female	WT	ND	ND	indel	indel	ND						○							○		○
#28	XY	Male	WT	indel	ND	indel	ND	indel										○			○		
#29	XX	Female	WT	indel	indel	indel	ND	WT													○		○
#30	XX	Female	WT	indel	ND	ND	indel	ND															
#31	XY	Male	WT	indel	indel	ND	WT	WT															
#32	XY	Male	WT	indel	ND	ND	ND	ND															
#33	XX	Female	WT	indel	ND	ND	indel	WT						○						○		○	
#34	XY	Male	WT	ND	ND	ND	ND	WT							○								
#35	XX	Female	WT	indel	indel	indel	indel	WT															○
#36	XX	Female	WT	indel	indel	ND	ND	ND						○		○		○		○			
#37	XY	Male	WT	indel	indel	ND	indel	WT								○		○					
#38	XX	Female	WT	indel	WT	indel	indel	indel						○									
#39	XX	Female	WT	indel	indel	indel	WT	indel										○			○		
#40	XX	Female	WT	indel	ND	ND	indel	ND							○					○			

Columns below 'Indel' and 'Deletion' show pairs of PCR primers used for genotyping.

Parentheses designate deleted regions.

WT: no mutation observed; indel: indel was observed; ND: not detected by PCR amplification; ○: PCR product was identified; Blank: PCR product was not identified

*PCR amplicon was not observed by any combinations of genotyping primers.

Table S2. Genotypes of F_0 mice obtained by injection of sgRNA2/6/7.

Mouse ID	Karyotype	Phenotypic sex	Indel			Deletion		
			F1/R1	F2/R2	F6/R6	F1/R2 (Δ7-2)	F2/R6 (Δ2-6)	F1/R6 (Δ7-6)
#41	XY	Male	indel	indel	indel	○		
#42	XY	Male	indel	indel	indel		○	○
#43*	XX	Female	ND	ND	ND			
#44	XX	Female	ND	ND	indel	○		
#45	XX	Female	indel	indel	indel			○
#46	XY	Female	ND	ND	indel	○		○
#47	XY	Male	indel	indel	indel			
#48	XY	Female	ND	ND	indel	○		○
#49	XX	Female	indel	ND	ND	○		○
#50	XY	Male	indel	indel	indel			
#51	XY	Male	indel	indel	indel			○
#52	XX	Female	ND	indel	indel			
#53	XX	Female	indel	ND	ND		○	
#54	XY	Female	ND	ND	ND			○
#55	XY	Male	indel	indel	indel			
#56	XY	Male	ND	indel	indel			
#57	XY	Male	ND	ND	indel	○		

Columns below 'Indel' and 'Deletion' show pairs of PCR primers used for genotyping.
Parentheses designate deleted regions.
WT: no mutation observed; indel: indel was observed; ND: not detected by PCR amplification; ○: PCR product was identified; Blank: PCR product was not identified
Mice exhibiting XY sex reversal are highlighted in yellow.
*PCR amplicon was not observed by any combinations of genotyping primers.

Table S3. Germline transmission of serial deletion alleles.

sgRNA combination	F_0 mice	Genotype of F_0	No. of F_1 genotyped	No. of F_1 mouse	
				WT	Deletion*
sgRNA1/2/3/4/5/6	#6	Δ2-3/Δ4-6	7	4	**1 (Δ2-3)**, 2 (Δ2-3; Δ4-6)
	#9	Δ2-3/Δ3-6/Δ5-6	8	0	**3 (Δ3-6)**, 2 (Δ2-3), 3 (Δ2-3; Δ3-6)
	#16	Δ2-6/Δ2-4	9	1	**2 (Δ2-4)**, **5 (Δ2-6)**
	#21	Δ2-5	7	6	**1 (Δ2-5)**
	#25	Δ4-6	5	3	**2 (Δ4-6)**
	#26**	Δ2-5	9	0	2 (Δ2-5), **7 (Δ5-6)**
	#33	Δ2-3/Δ4-6	3	2	1 (Δ2-3)
	#34	Δ2-4	14	11	3 (Δ2-4)
	#35	Δ5-6	7	5	2 (Δ5-6)
	#37**	Δ3-4	11	5	6 (Δ2-5)
sgRNA2/6/7	#45	Δ7-6	9	5	**4 (Δ7-6)**
	#57	Δ7-2	10	6	**4 (Δ7-2)**

*Established deletion lines are shown in bold.

**Deletion patterns observed at F_1 generation was not identified in F_0 mice. #26 and #37 are considered as mosaic individuals.
